# Table Cleaning Task by Human Support Robot Using Deep Learning Technique

**DOI:** 10.3390/s20061698

**Published:** 2020-03-18

**Authors:** Jia Yin, Koppaka Ganesh Sai Apuroop, Yokhesh Krishnasamy Tamilselvam, Rajesh Elara Mohan, Balakrishnan Ramalingam, Anh Vu Le

**Affiliations:** 1 Engineering Product Development Pillar, Singapore University of Technology and Design (SUTD), Singapore 487372, Singapore; yin_jia@mymail.sutd.edu.sg (J.Y.); apuroopkgs@gmail.com (K.G.S.A.); rajeshelara@sutd.edu.sg (R.E.M.); 2 Department of Electrical Engineering, Clemson University, Clemson, SC 29631, USA; ykrishn@g.clemson.edu; 3 Optoelectronics Research Group, Faculty of Electrical and Electronics Engineering, Ton Duc Thang University, Ho Chi Minh City 700000, Vietnam

**Keywords:** inspection, table cleaning, deep learning, CNN, human support robot, food litter detection

## Abstract

This work presents a table cleaning and inspection method using a Human Support Robot (HSR) which can operate in a typical food court setting. The HSR is able to perform a cleanliness inspection and also clean the food litter on the table by implementing a deep learning technique and planner framework. A lightweight Deep Convolutional Neural Network (DCNN) has been proposed to recognize the food litter on top of the table. In addition, the planner framework was proposed to HSR for accomplishing the table cleaning task which generates the cleaning path according to the detection of food litter and then the cleaning action is carried out. The effectiveness of the food litter detection module is verified with the cleanliness inspection task using Toyota HSR, and its detection results are verified with standard quality metrics. The experimental results show that the food litter detection module achieves an average of 96% detection accuracy, which is more suitable for deploying the HSR robots for performing the cleanliness inspection and also helps to select the different cleaning modes. Further, the planner part has been tested through the table cleaning tasks. The experimental results show that the planner generated the cleaning path in real time and its generated path is optimal which reduces the cleaning time by grouping based cleaning action for removing the food litters from the table.

## 1. Introduction

Due to long working hours, low wages, unwillingness to work as a cleaner, workforce shortage has been a constant problem for food court cleaning and maintenance tasks in recent times [1]. Recently, many robotic platforms are designed for different cleaning application which include floor cleaning [2,3], facade cleaning [4], staircase cleaning [5], pavement cleaning [6,7] and garden cleaning [8]. However, these robot architectures could not support table cleaning and maintenance tasks. In this context, HSR can be a viable candidate for this task [9,10]. However, configuring HSR for cleanliness inspection and food litter collection is a challenging task [11]. Because, the robots need optimal food litter (food scraps, stains, spillage) detection system and real-time planner algorithm to execute the cleaning and inspection task [12,13].

Various techniques have been developed for cleaning robots to recognize the different class of litter (garbage, dirt, liquid spillages, and stains) and compute the cleaning strategy. Among them, computer vision-based techniques are widely used in cleaning robots for recognizing the litter and compute the cleaning action [14,15,16,17,18,19]. Andersen et al., built up a visual cleaning map for cleaning robots using a vision algorithm and a powerful light-transmitting diode. The sensor recognizes the grimy region and generates the dirt map by examining the surface pictures pixel-by-pixel utilizing the multi-variable statistical method [15]. David et al., proposed high-level manipulation actions for cleaning dirt from table surfaces using REEM a humanoid service robot. The author uses a background subtraction algorithm for recognizing the dirt from the table and Noisy Indeterministic Deictic (NID) rules-based learning algorithm to generate the sequence of cleaning action [16]. Ariyan et al., developed a planning algorithm for the removal of stains from non-planar surfaces where the author uses a depth-first branch-and-bound search to generate cleaning trajectories with the K-means clustering algorithm [17]. Hass et al., demonstrated the use of unsupervised clustering algorithm and Markov Decision Problem (MDP) for performing the cleaning task where unsupervised clustering algorithm is used to distinguish the dirt from surface and MDP algorithm is used to generate the maps, and transition model from clustered image is used to describe the robot cleaning action [18]. Nonetheless, these approaches have some practical issues and disadvantages for using in food court table cleaning; the detection ratio relies heavily on the textured surfaces, which makes it challenging to identify the litter type as solid or stain or liquid spillage [12,20,21]. The litter classification is a crucial function for food court table cleaning and inspection using the mobile service robot. It will play a significant role in finding the cleaning mode, generating the cleaning path, and inspecting the cleanliness of the table [13].

Deep learning-based object detection is an emerging technique. Deep Neural Network architecture can be modified and optimized to solve different complex tasks in computer vision such as object classification, object detection, and object segmentation applications. It has been widely used in the robotic filed to detect the obstacles [22], pick and place the objects [23], monitoring construction sites [24] path planning [25]. Recently, the cleaning robot application uses deep learning algorithms for recognizing the various class of litters and generate the cleaning strategy. Fulton et al., use deep-learning systems for autonomous submerged automobiles for marine debris detector. The author realizes that Convolutional Neural Network (CNN) and Single Shot MultiBox Detector (SSD) are more accurate in comparison with YOLOv2 and Tiny YOLO frameworks [26]. Rad et al. [27], trained the overfeat-googlenet to recognize the outdoor debris. The authors utilized 18,672 pictures of different kinds of litters and squanders to prepare CNN for recognizing the solid trash, for example, leaves, papers, nourishment bundles, jars, and so forth in the outdoor environment. In Reference [28] Jiseok et al., proposed contaminant-detection machine-vision system for façade cleaning robot where the author uses the YOLOv3 object detection framework with hue, saturation, value (HSV) color space and grayscale algorithms for detecting the object-type contaminant, area-type contaminants and rust particle-type contaminant on the facade. Through Taguchi optimization method, the author improves the detection robustness of the model under various height and brightness conditions. In Reference [13], the author proposed a machine learning technique for the detection and classification of debris using the MobileNet v2 SSD CNN framework for the classification of solids and liquid spillage debris and the support vector machine (SVM) classification for the size of liquid spillage. However, this work does not describe the cleaning strategies. Chen et al. [29] implemented a computer vision-type robot capture system for automatic trash sorting where Fast Region Based Convolutional Neural Network (Fast R-CNN) is used to monitor various objects in the scene.

Developing a real-time planner algorithm is another critical challenge of cleaning robot applications. In the planner, module path planning is a key component that plays the vital define the cleaning path according to food litter detection. In literature, various methods are available for autonomous cleaning robots for solving the path planning problems such as Dijkstra’s algorithm, D-star, A-star, rapidly exploring random tree (RRT), and probabilistic roadmap (PRM) technique. The pros and cons of each scheme and path smoothing techniques for autonomous Robot Navigation have been described in Reference [30]. Because of the complexity of A-star, D-star, these algorithms are mostly used in dynamic environments. On the other hand, the PRM scheme has more suitable for static environments. As a consequence, the PRM algorithm is applied for the table cleaning task where the objects of interest stay static. Furthermore, positional information, muscle stiffness of the human arm, contact force with the environment also play important roles in understanding and generating human-like manipulation behaviors for robots as in Reference [31].

Motivated by the works mentioned above, this work proposes the deep learning-based food litter detection system and a path planner algorithm for the Toyota Human Support Robot (HSR) [10,32] to accomplish the table cleaning and inspection task for the standard food court setting. The technical contributions of the paper are as follows.

(1) The 2D location from the output of the lightweight Deep Convolutional Neural Network (DCNN) based litter detection model is combined with the depth data to yield the 3D location of the food litter on top of the table.

(2)The proposed framework can provide a high confidence level in classifying various types of litters like liquid, solid.

(3) The planner algorithm computes the cleaning mode and find the cleaning path for removing the food litters from the table. The planning and execution behaviors of how the robot cleans the dirty table are inspired by the actual cleaning activities conducted by the human. The real-time planner uses the Depth-First Search (DFS) [33] and Probabilistic Road Map (PRM) [34] algorithm, which are unified for generating the cleaning path. The DFS technique generates the initial path map for collecting food litters from the table, and the PRM scheme optimizes the path according to the motion planning function.

(4) The proposed method is validated in Toyota HSR robot and its efficiency has been verified with standard quality metrics.

The rest of the paper is organized out as follows: Section 2 describes the litter detection framework and planner module. Section 3 describes the experimental setup and the experimental results. Conclusions and future work are finally presented in Section 4.

## 2. Proposed System

**Figure 1:**
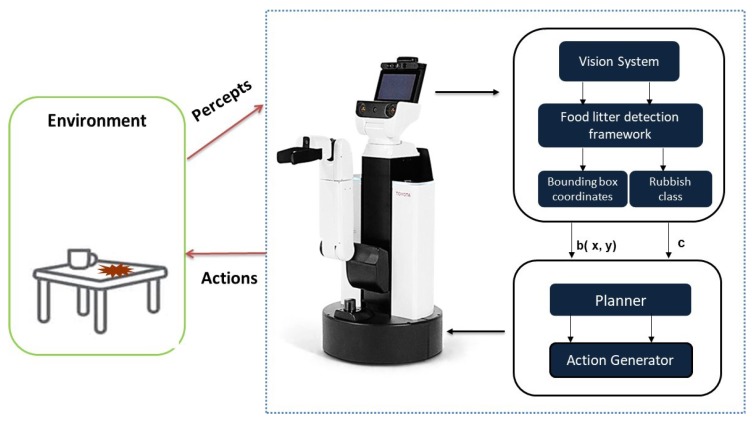
Block diagram of proposed scheme.

Figure 1 shows the functional block diagram of proposed scheme. It comprised of two modules, include DCNN based litter detection framework and planner module. The detection model comprises of two parts—a feature extractor and a bounding box predictor. Here, the feature extractors extract the specialized features pertaining to a litter classification then generates the feature map. As a consequence, bounding box predictors locate the objects in an image and distinguish the litter class (c) using feature maps extracted by the feature extractor. The detected boundary box region coordinates b(x,y) is further converted to 3D coordinate in the world frame in meters from the robot base frame. Then 3D coordinates of the center of the boundary box become the representative for each object of interest, which is used to control the HSR arm actuator. The detailed description of each module is described as follows.

### 2.1. CNN Based Litter Detection Framework

**Figure 2:**
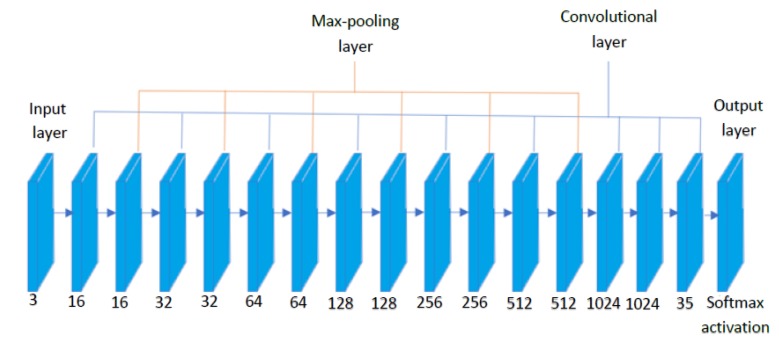
Convolutional Neural Network (CNN) architecture.

The proposed CNN network contains 16 layers with 9 convolutional layers and 6 pooling layers. The network is built on python with DarkFlow as a backend. DarkFlow is an open-source object detection algorithm that can be used for object detection and localization. The advantage with DarkFlow is that the architecture of the network can be altered, that is, changing the activation functions, network layers, and training using custom objects. The number of convolutional and pooling layers has been decided to make sure that the network does not cause any overfitting. The huge number of hidden layers could, in turn, cause overfitting issues. We started with adding convolutional and hidden layers until we receive a good enough F-1 score [35]. Figure 2 shows the functional block diagram of litter detection architecture and detail of each layer include filter size, padding, stride are given in Table 1.

#### 2.1.1. Convolutional Layers

Convolutional layers are used to extract higher-level features that could be used for performing some complicated classification. In a nutshell, the convolutional layer performs the operation of the convolution between its input and filter of the desired size. The number and size of the filters are given by the user as a parameter to the layer. The Equation (1) describing the operation of convolution function.

(1) $$\left(f*g\right)\left(t\right)=\int_{-\infty }^{\infty }f\left(\tau \right)\;g\left(t-\tau \right)d\tau$$

where, *f* and *g* are two variables that are involved in convolution. *f* is the input and *g* is the filter function.

The output is a two-dimensional activation map that provides the response of convolution at each spatial position. Based on the size of the filter and the size of the input, the size of the output can be determined using Equation (2).

(2) $$outputsize=\frac{\left(I-K+2P\right)}{S}+1$$

where, *I* is input image matrix, *K* is the filter size, *P* is zero padding and *S* is the stride length.

The first convolutional layer takes in images of size 416× 416 × 3. The number of channels in the filter of a convolutional layer must be equal to the number of channels in the output of the previous layer.

#### 2.1.2. Pooling layer

Pooling layers are generally designed to prevent over-fitting and, by applying non-linear down-sampling, it reduces the size and robustness to expedite computation. Zero-padding is not performed within the pooling layer as it would work against the purpose of the layer. The predominantly used pooling methods are:

- Max pooling
- Average pooling

Max pooling: The maximum adjacent value is taken from the input image based on the position of pixels. This is implemented through input channels. Median pooling: The average of all values within the region covered by the filter is taken and assigned as the output value. The other important settings for the network layers are the hyper-parameters, which were set as follows:

- Batch size = 64
- Learning Rate = 0.001
- policy = steps
- Threshold = 0.5

#### 2.1.3. Bounding Box

In this work, we are focusing on litter object localization with class. Specifically, the customized CNN network will give the exact location or Region Of Interest (ROI) in the color image. The location of the litter object is wrapped inside the bounding box. The idea behind the bounding box is that each image is divided into segments with an identical area, and a target vector has been generated for them (Equation (3)). The bounding box target vector for training would be as follows.

(3) $$y=\left[\begin{array}{c} P\\ X_{min}\\ Y_{min}\\ X_{max}\\ Y_{max}\\ C_{1}\\ C_{2} \end{array}\right]$$

where *P*—binary value which determines if there is an object of interest in the image, $X_{min}$—Upper left x Bounding Box Coordinate, $Y_{min}$—upper left Y bounding box coordinate, $X_{max}$—Lower right x bounding box coordinate, $Y_{max}$—Lower right y bounding box coordinate, $C_{1}$ is 1 if the object belongs to class 1, else zero, $C_{2}$ is if the object belongs to class 2, else zero. Further, Intersection Over Union (*IOU*) method (Equation (4)) is used to remove any overlapping bounding boxes and to measure the accuracy of the bounding box with respect to the ground truth. *IOU* is the ratio of the area of overlap to the area of union.

(4) $$IOU=\frac{\left(A\cap B\right)}{\left(A\cup B\right)}$$

For fixing the various *IOU* thresholds were tested out during the experiments, and the best one has been picked. In our case, we have chosen the *IOU* threshold to be 0.5, which has been widely reported in the literature and provide more stable results. This means when evaluating the output; if the *IOU* calculated from the predicted bounding box and the actual bounding box is equal to more than 0.5, we would consider that as a correct output, whereas anything below 0.5 is considered as the wrong predictions of the bounding box coordinates. The average *IOU* matching calculated over all the bounding boxes in the test set is 0.7044, with an overall confidence 0.589.

### 2.2. Planner

**Figure 3:**
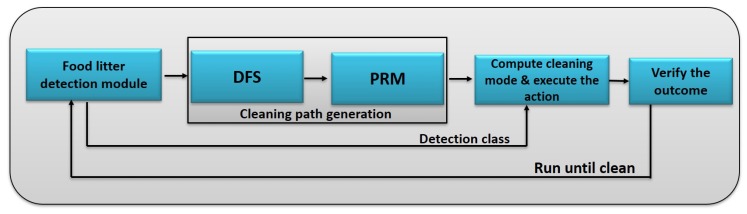
Planner process flow diagram.

The planner module is developed for accomplishing the table cleaning task through HSR. The planner has two functions, namely finding the cleaning method and constructing the cleaning path. Figure 3 shows the process flow diagram of the planner module. It uses the litter detection framework for finding the cleaning method and constructing the cleaning path.

**Figure 4:**
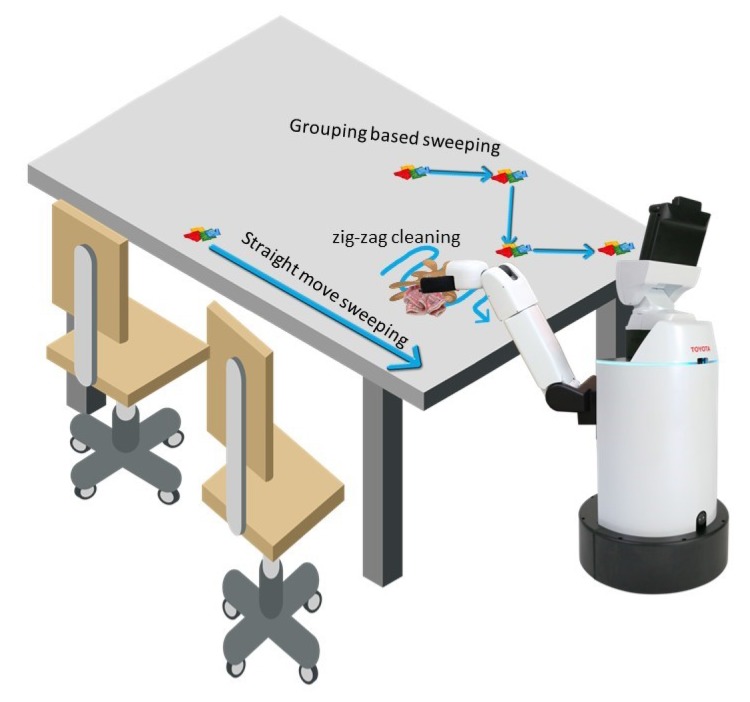
Planned Cleaning action.

Two cleaning methods are adopted for the table cleaning task, which includes sweeping and wiping, where the sweeping method is used to clean the solid and semi-solid food litter, and wiping mode is used to remove stains. Straight move or grouping based sweeping action is adopted for cleaning solid, and semi-solid food litter where grouping based sweeping is used for cleaning the multiple litters in one shot and straight move is used for removing the separated food litters. Further, zig-zag cleaning action is considered for wiping the stains. Figure 4 demonstrate the three different cleaning action.

#### Construction of Cleaning Path

To construct the path, the planner uses the probabilistic road map algorithms. Here, the DFS technique generates the initial path from the detected bounding region, then PRM scheme has been applied for fine-tune path according to HSR mobility function. The key element of path planning function $P=(nodes,D_{goal}\left(x,y\right),edges)$. Here, the detected bounding box are considered as nodes $N=(b_{1}\left(X,Y,d\right),b_{2}\left(X,Y,d\right),\ldots ,\left(b_{n}\left(X,Y,d\right)\right)$, $D_{goal}\left(x,y,d\right)$ is the robot dust collection point or edge of the table and *P* is the feasible path for connecting the nodes. The path planner constructs two-way path, such as direct path planning and grouping based path planning.

Initially, the planner starts the path planning by exploring the path which starts from the leftmost upper node, and that node is marked as $q_{start}$. The DFS scheme has been used to explore the path between $q_{start}$ and remaining nodes. It follows the vertical path searching, and it starts with the first neighbor and continues down the line as far as possible. Once it reaches the final node, then it backtracks to the first node, where it was faced with a possibility to change the next neighbor. Here, the backtracks nodes are marked as connection point of $D_{goal}\left(x,y,d\right)$. Once DFS explores the path. PRM has been adopted to post-processing the path that fine-tune the cleaning path according to HSR mobility function. The post-processing steps first connect the backtrack point with $D_{goal}\left(x,y,d\right)$ and generate the new path. In addition, analysing the distance of each connected node in the generated path used Equations (5) and (6). As long as the distance between the two consecutive nodes in the path is less than or equal to a predefined threshold (maximum arm reachable distance), the node considered as neighbor and path is valid. Otherwise, a node has been neglected, and the new pathfinding process has been initiated for the neglected node. The process repeats for all neglected nodes. The Algorithm 1 outline the path planning scheme.

(5) $$Nq=\{q^{\prime }\in N|D\left(q,q^{\prime }\right)=sqrt\left({\left(X_{q}-X_{q}^{\prime }\right)}^{2}\right)\leq D_{max}\},$$

where $D_{max}$ is threshold, *q* is new generated node, $q{}^{\prime }$ is neighbor node, and D(q,q′) is Euclidean distance Equation (6).

(6) $$D\left(q,q^{\prime }\right)=∥q-q^{\prime }∥.$$

**Algorithm 1** Cleaning Path Generation
1:
**Step 1**
2:*N* is an array holds all the nodes position3:*P* is an array used to store the cleaning path4:$q_{start}$ = leftmost(*N*);     Set uppermost left nodes as $q_{start}$5:load $q_{n}$ = N−16:$q_{goal}$ = def_goal(litter collection point);   the function load the litter collection point as $q_{goal}$7:
**Step 2**
8:**while** cleaning path between $q_{start}$ to $q_{n}$ not discovered **do**9: ***Q*, backtracknode, internodesandPath** are variable used for store the temporary value10: **for** $n=0;n<q_{n};n++$ **do**11:  Q= dfs_paths($q_{start}$, $q_{n}$);        Explore the path between $q_{start}$ and N−1 nodes12: **end for**13: backtracknode = **backtrack.find**(Q);     the function find the backtrack node in the explored path14: internodes = **group.find**($q_{start}$, backtracknode);     the function collect intermediate node between $q_{start}$ and backtracknode15: Path = def_PRM_node_connect($q_{start}$, internodes, backtracknode, $q_{goal}$, *R*);   PRM function connect the nodes according to threshold function R16: **plt.plot** (Path)17:
**end while**
18:**visited.add(Path)**;   Mark all path identified node in *N*19:Store the path in **P**20:load $q_{n}$ = N−Path;   exclude path generate node from N21:**set.new**($q_{start},N$);   set new $q_{start}$ from updated N22:Run the **Step 2** up-to generate the path for remaining nodes


## 3. Experimental Setup & Results

This section describes the experimental setup and experimental results of the proposed scheme. There are three phases in this section which including configuring algorithms in HSR, training data preparation and validation, and evaluating the table cleaning and inspection with HSR.

### 3.1. Configuring HSR

**Figure 5:**
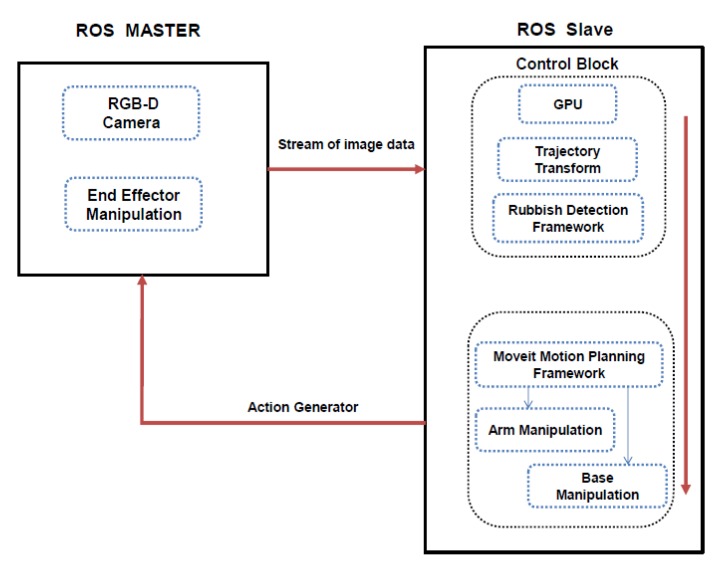
Human Support Robot (HSR) hardware architecture configuration.

Toyota HSR is utilized in our experiment to test the proposed scheme. The contour structure of the HSR robot is shown as in Figure 1. There are five key modules in the HSR robot, including an RGB-D camera in the head region to perceive the objects in the environment, 4 degrees of freedom arm manipulator with a capacity of flexible manipulation enabling it to reach all the points in the 3D space. The proposed system is built on the Robot Operation System (ROS) platform [36]. ROS provides the infrastructure and mechanism that enable hardware components to work smoothly together. The ROS master installed on the main system monitors the entire ROS system. The ROS topics transmitted in the ROS network through local connection or data networks enable the communication between ROS nodes. The 3D robot coordinate frames such as the base movement of the robot, RGB camera, base, arm, grabber are maintained by transformation frame service in the ROS system. By referring to the lookup table in the transformation frame, HSR understands the relative 3D offset between the pair of these frames to control the corresponding actuators. Figure 5 shows the schematic representation of HSR hardware architecture configuration, which comprises of two computing units, namely primary and secondary systems. The primary system contains master ROS which has access to the sensor data and the motion planner framework. Here, the primary system is configured to perform the path planning and arm manipulation task. The primary system uses the MoveIt open-source software framework application program interface (API) to control and plan the motion of the robotic arms.

The secondary system is Nvidia’s Jetson TK1 board (GPU), operating as a ROS slave. The slave block contains a control block and a litter detection framework. The control block governs the interface between the deep learning framework and ROS. It wraps the data received from the deep learning framework into the ROS compatible messages and sends it to the primary system. Similarly, it unwraps the ROS message into a suitable format that is required by the deep learning framework. Both the primary and the secondary systems are connected over Transmission Control Protocol/Internet Protocol (TCP/IP) and share all the ROS topics.

To execute the cleaning task, the primary system enables the RGB-D sensor and collect the RGB-D stream of image data to the secondary system. The GPU in the secondary system, which is running the deep learning framework, takes in the input data and performs the object detection and classification task and returns detected information, which contains the coordinates and types of litters.

Since the boundary box of the detected object is in the image coordinate frame, the knowledge of the point in the 3D world coordinate system is essential for the robot to manipulate the arm and grabber. The pixel coordinates in the color image of the boundary box, including the center, top left, bottom left, and top right, are converted to 3D coordinate in the world frame in meter from the robot base frame. Then the 3D coordinate of the center of boundary box becomes the representative for each object of interest. In this paper, we assume that the RGB-D camera is set up so that its Field of View (FOV) covers the whole table, which needs to be cleaned. To do the 2D to 3D conversion, projective geometry is used for mapping the point in the image plane to 3D point in the world frame. Intrinsic and extrinsic parameters of the camera are estimated by the camera calibration method [6]. Translation matrix $T=[t_{1}\;t_{2}\;t_{3}]$ is assumed from perception system after calibration from the origin of world coordinate and orientation matrix R=[roll,pitch,yawn]. Camera intrinsic parameters lies in *K* with focal length $f_{x},f_{y}$, principle point $\left(x_{c},y_{c}\right)$, pixel size (sx,sy) and distortion coefficients σ, any pixel $p=[p_{x}\;p_{y}]$ on the image plane with 3D coordinate W=[XYd] world plane and W can be calculated by p=HW where H=K[RT] is the homogeneous matrix. After identifying the point in the 3D world coordinate frame and assigning it to a specific object frame, the transformation frame service of ROS will maintain the relative location between this point to robot coordinate frames.

Upon receiving the control information, the primary system then generates the cleaning map for executing the cleaning action through arm motion. After the path has been derived from DFS and PRM techniques, then Moveit [37] service of ROS has been used to path following, arm controlling, and base motions which have been optimized by Toyota for HSR. Specifically, by considering robot kinematics, curvature continuities of the robot arm, path feasibility, Moveit executes the robot arm trajectory to avoid smooth sudden stops and collisions with fragile objects. Upon executing the motion plan to clean the litters, the arms return to its home position. After visiting all the litters locations at least once, the primary system requests the secondary system (GPU) for a confirmation of the cleaning by sending in the RGB-D image data. If the food litters are still present, then the secondary system will request the primary system for another round of cleaning.

### 3.2. Training Data Preparation and Validation

The HSR RGB-D camera is used to capture the litter data set. The specification of RGB-D camera is given Table 2. Images are collected from the robot perspective with a different angle. In total, there are 3000 images captured at different table backgrounds with various types of food litters, include food scrub, liquid spillage, and stains.

In addition to that, the CNN learning rate was improved, and over-fitting was prevented by applying data expansion to the captured images. Data expansion applies simple geometrical operations on images like scaling, rotating, shifting, and flipping to increase the number of samples in the data set. Further, the images are resized to 416×416 pixels to reduce the computational time for training. Furthermore, the dataset is labeled as two classes that include solid and liquid. The dataset was divided into two classes, with 1500 images per class. One class of image contains only a single type (solid or liquid) food litters. The other one consist of mixed food litter in the same image. The CNN network was developed in the Tensorflow framework and trained in Intel Xeon E5-1600 V4 CPU with 64 GB RAM and an NVIDIA Quadro P4000 GPU with 12 GB Video memory.

K-fold cross-validation is adopted in this work to validate the dataset. In this method, the dataset is divided into k subsets, and K-1 is used for training. This work uses 10 fold cross-validation. The images shown are obtained from the model with the highest accuracy.

### 3.3. Evaluate the Table Cleanliness Inspection with HSR

**Figure 6:**
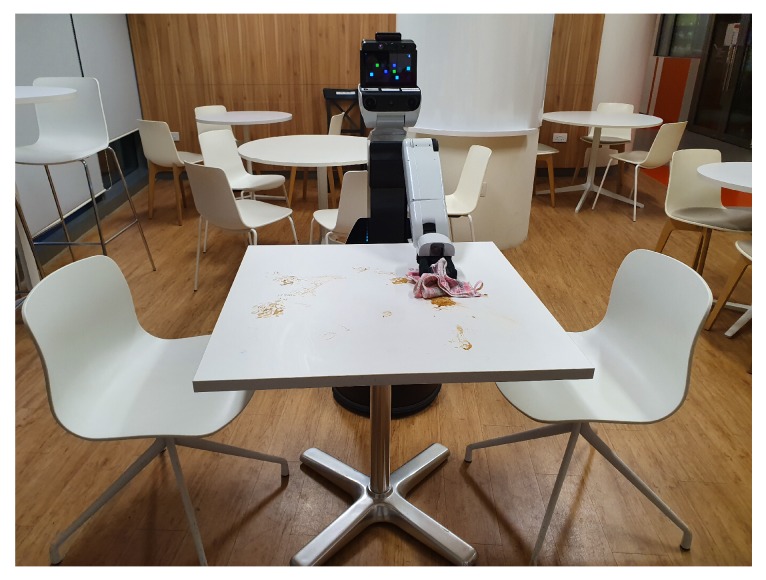
Experimental test bed.

The cleanliness inspection part has been assessed through food litter detection on the dining table. The video demonstrating how HSR detects litters then executes the cleaning actions are provided as Video S1 in Supplementary Material. This is a crucial component of the proposed scheme. Hence, the performance of the robot was ensured for the accuracy of the detection model. To carry out the first experiment, the trained model was configured in HSR secondary system. The experiment was tested in square and circular dining tables, which are arranged like a food court dining pattern, as shown in Figure 6. After configuring the algorithm in hardware, the robot is placed in a work-space. For experimental purposes, various food litter (solid food class, stains, and spillage) are scattered on the table looks like unclean table, and detection has been analyzed through a remote console.

**Figure 7:**
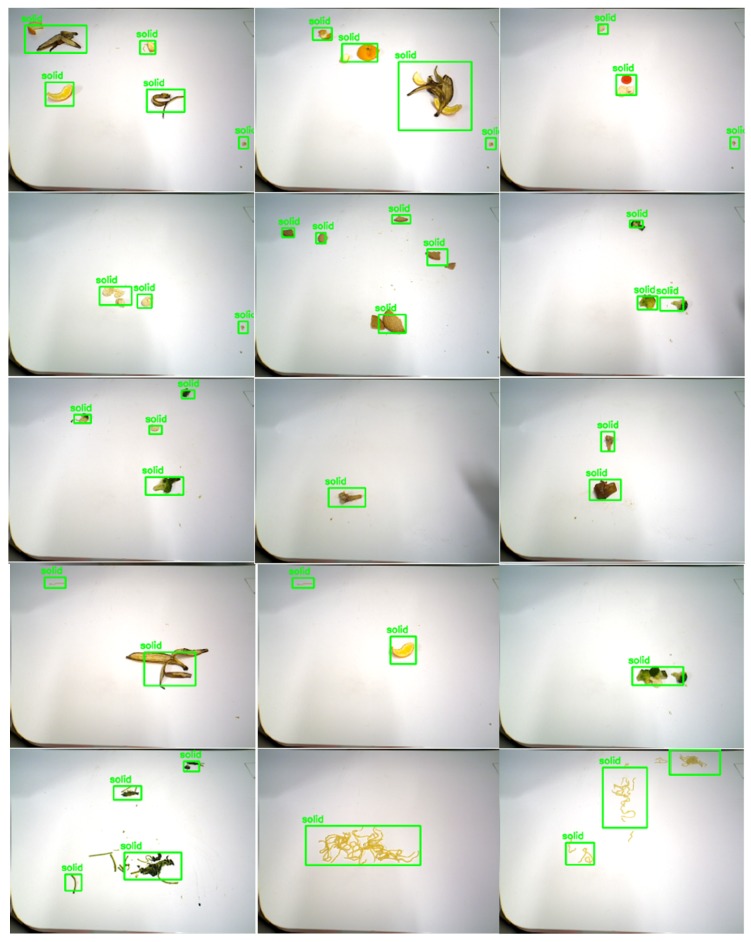
Solid litter detection results.

**Figure 8:**
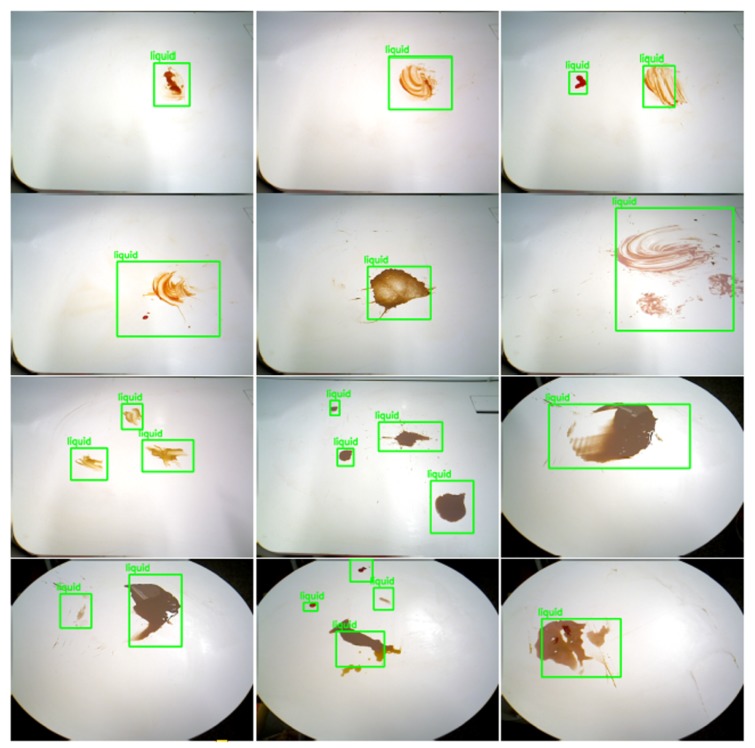
Liquid spillage and stains detection results.

**Figure 9:**
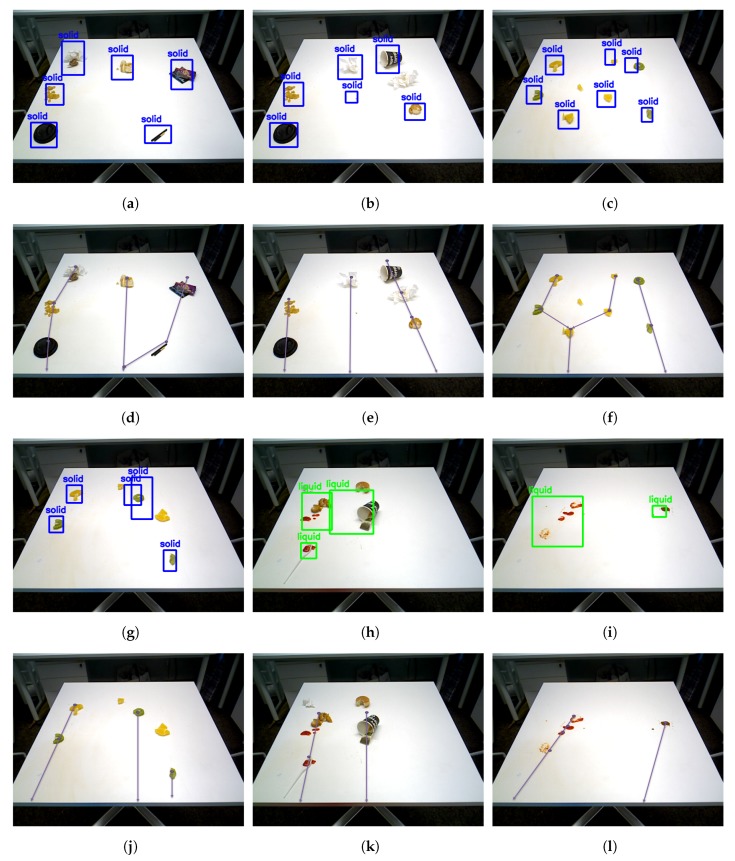
(**a**–**c**,**g**–**i**): Food litter detection results, (**d**–**f**,**j**–**l**): Cleaning path map for detection results.

Figure 7 and Figure 8 shows the detection results for different types of food litter captured by HSR in different angles. The results ensure that the performance of the developed litter detection framework can detect most of the food litters includes solid class foods, stains, and liquid spillages on the dining table. Solid food litter detected typically has a 97% or higher confidence level and stains, and liquid spillage has been recognized at 96% or higher confidence level, respectively. Further, the miss rate (Equation (7)) and false rate (Equation (8)) metric [13] are evaluated for the proposed litter detection framework. These two scenarios can be better understood by observing the Figure 9b,c,h. In Figure 9b,c are examples of miss detection, where some food litter is not detected by the model. Figure 9h is an example of false detection where the solid type food liters are detected as the liquid class. Here the miss rate represents the case, where target litter is not detected by the model from given input image set. Whereas, false positive indicates that the type of litter (solid, liquid etc.) is wrongly detected from the input image set. In our model, the overall miss rate and false rate is less than 3% and 2% respectively for an input image set comprising of hundred solid and liquid food litter objects.Since the depth features of the solid and liquid litter of trash are not so obvious and are affected by depth sensor noise, perspective viewpoint, depth data alone is not sufficient. In addition, using only the depth is hard to identify litter from other solid and liquid objects such as food or items on the table because they have the same depth features as litter. As a consequence, the DNN network needs to be customized to give the acceptable solid/liquid classification on the table with only depth information. We will consider using depth data directly for classification in the future works

(7) $$\eta_{miss}=\frac{n_{missnum}}{n_{testset}}\times 100\%$$

(8) $$\eta_{false}=\frac{n_{falsenum}}{n_{testset}}\times 100\%$$

where $n_{testset}$ is total number of test objects.

### 3.4. Comparison with Existing Schemes

This section describes the comparative analysis of proposed work with other existing case studies in the literature. The comparison analysis has been performed based on CNN and non-CNN methods used on different cleaning robot applications for the detection of different kinds of litter ( dirt, garbage, marine debris). Table 3 shows the comparison with non-deep learning based approaches. Table 3 results indicate that non-deep learning based schemes detection accuracy is average of 80%, and detection accuracy heavily relies on background surface and brightness of the litter objects which lead to false detection of objects. However, the authors describe that false detection can be overcome by re-scaling the filter response manually. Further, models are able to detect the litter based filter function and cannot classify the litter classes, which is the fundamental limitation of non-deep learning based litter detection model.

Table 4 shows CNN case study and Table 5 shows comparative analysis of CNN based litter detection schemes, which are implemented using various CNN based object detection schemes. Generally, the object detection framework efficiency has been assess through accuracy, precision, recall, and F-1 score, miss rate and false rate metric [13]. In literature point of view, compare our proposed method with the other litter detection frameworks is very hard, because each model uses a different CNN topology and training parameters. Also, the data-sets are different. Hence, we resort to describing the essential characteristics of each model and enlist its pros and cons for the common attributes.

In the literature, Faster RCNN with ResNet or inception, Mobilnet V2 SSD [42], and YOLO object detection framework are widely reported framework for litter detection. The table results indicate that faster RCNN- resnet or faster RCNN-inception framework has higher accuracy compared to other models. However, faster RCNN requires tremendous computing resources due to its region-based convolutional, which makes it less suitable to be deployed in low computing devices. At the same time, SSD-MobileNet based implementation provides a right balance between accuracy and computational time where the SSD mobilenet model uses a defined set of sizes for the bounding boxes and uses the more efficient depthwise convolution layers which significantly reduces the computation time and inference time. YOLO v2 is much faster than SSD Mobilenet, which is built on a dark flow framework and uses a single convolutional network to predict the bounding boxes and the class probabilities for these boxes. Hence, it required less computation power and low inference time than other models. Furthermore, our framework follows the YOLO dark flow; hence it achieves considerable accuracy and low computation time similar to the YOLO model. YOLO is prone to overfitting, and therefore, we have set the learning rate to be much less than many other previous works. Further, we have also limited the number of hidden after the convolutional and Max pooling layers. Finally, we have made sure that the training data is not skewed that is, the number of training data in each of classes is pretty much equivalent for training.

In contrast to a non-deep learning model, Deep learning techniques offer a significant advantage in scenarios when sufficient data is available. These techniques are able to autonomously extract features from images, which allow them to learn features and patterns which are difficult to figure out statistically. CNN architectures are specifically good for extracting features from images since they use a combination of pixels next to each other for features.

### 3.5. Validate the Planner Module

**Figure 10:**
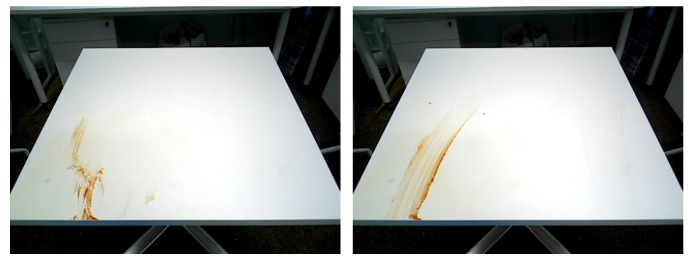
Outcome of cleaning task for stains and spillages.

**Figure 11:**
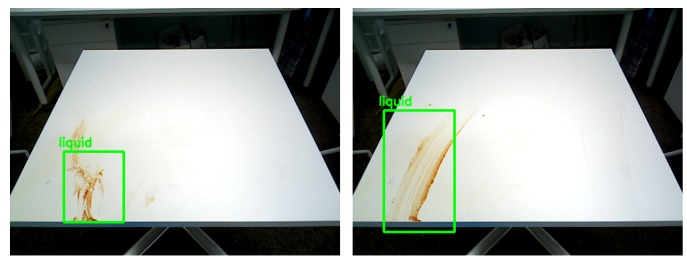
Cleanness inspection after execute the cleaning task.

The efficiency of the planner module has been tested through cleaning path generation and table cleaning tasks using generated cleaning path. Two experiments are conducted to assess the table cleaning function. In the first experiment, Robot cleans the solid food litter on the table. The second experiment is set to clean the presence of both solid waste and liquid spillages. To carry out the experiments, firstly, the litter detection module was run to recognize the food litter, then the planner was executed to generate the cleaning path. Figure 9 shows the litter detection results and their respective cleaning path for two experiments. The experimental results indicate that the path planner covers most of the detection region efficiently. Further, the generated path was loaded into the arm and base motion planning module to execute the cleaning task. The outcome of the cleaning task was verified after executing all the cleaning path. Figure 10 shows the outcome of the task after executing the cleaning task. Here, the stains and liquid spillage litters were spread when the robot tries to clean. So, those litter regions need to do multiple rounds of cleaning action and cleanliness inspection, as shown Figure 11 (cleanliness inspection after executing the cleaning task). Further, the solid food litter was cleaned in one iteration. However, some of the litter’s regions are not able to clean in the first iteration because some food litter is scattered, and some of the litter regions are not able to cover accurately due to noise of depth data, which affect the process of 3D coordinate into the world coordinate.

The Table 6 shows the average computation time for detection, path planning, and motion planning. The planner part runs on the CPU and takes about an average of 0.0145 seconds. Further, for cleaning the three to five food litters, the execution module takes approximately an average of 210.5 seconds.

The test results indicate that the proposed algorithm can satisfy the real-time requirement. It is more suitable for executing the cleanliness inspection and table cleaning application using HSR.

### 3.6. Practical Limitations

In this work, the cleaning actions depend upon the precise value of the depth information acquired by the RGB-D cameras, which have a restricted resolution. Even though the use of cloth has some advantages, the expected movement of the litter is not reached by the actions. Further, The Toyota arm has 5 degrees of freedom rotation, because of which the arm planning becomes complicated by involving the robot base to reach certain points on the table. This can be solved by adding an additional degree of freedom to the robotic arm. In our feature work, we plan to improve the quality of the RGB-D vision system and HSR motion planner function, which helps to predict the object position and orientation accurately and improve the cleaning efficiency. The computing power of the existing CPU installed on the HSR is relatively slow, an extra computing board with additional hardware computing power can be used to expedite the detection and identification of food litter.

## 4. Conclusions

Developing a cleaning robot platform should have two critical features. The environment should be of real-world setting, and the platform should be able to reach every nook and corner of the cleaning region. This proposed cleaning platform Toyota HSR is developed for assisting humans in a general setting, and proven to be efficient in conventional environments. The proposed framework is tested on a common food-court like setting so that the real-world implementation can be without any issues. Unlike the earlier results, the proposed work focuses on classifying the litter into various types, for which the cleaning process is different. Moreover, the classification module is augmented with an optimal path planning module and control module to achieve high efficiency. The efficiency of the detection framework is evaluated through cleanliness inspection, and its detection accuracy was measured through standard quality metrics. The experimental results show that the proposed cleaning framework detects most of the food litter with highest detection accuracy among the considered methods, and HSR cleaning part takes an average of reasonable time to clean the three to five food litters on the table. The proposed method can be used to clean vertical surfaces like glass panels, windows in homes, among others. The module can be used to identify litters not only on tables but also on the floors. This scaling of this application can open new doors in the cleaning industry.

## Figures and Tables

**Table 1:** Convolutional Neural Network (CNN) layer description.

Layers	Filter Size	Padding	Stride	Number of Filters
Convolutional layer 1	416	same	1	16
Max Pooling layer 1	208	same	2	16
Convolutional layer 2	208	same	1	32
Max Pooling layer 2	104	same	2	32
Convolutional layer 3	104	same	1	64
Max Pooling layer 3	52	same	2	64
Convolutional layer 4	52	same	1	128
Max Pooling layer 4	26	same	2	128
Convolutional layer 5	26	same	1	256
Max Pooling layer 5	13	same	2	256
Convolutional layer 6	13	same	1	512
Max Pooling layer 6	13	same	1	512
Convolutional layer 7	13	same	1	1024
Convolutional layer 8	13	same	1	1024
Convolutional layer 9	13	same	1	35

**Table 2:** RGB-D camera specification.

Specification	Details
Dimensions	18 × 3.5 × 5
Resolution	SXGA (1280*1024)
Field of View	58${}^{{}^{\circ}}$ H, 45${}^{{}^{\circ}}$ V, 70${}^{{}^{\circ}}$ D (Horizontal, Vertical, Diagonal)
Distance of Use	Between 0.8 m and 3.5 m
Power Consumption	Below 2.5 W
Frame Rate	30 fps

**Table 3:** Non-Deep Learning Based Detection.

Case Study	Algorithm	Detection Accuracy
Floor cleaning: Dirt and Mud detection on floor [14]	Spectral residual filter	75.45
Floor cleaning: Dirt and mud detection [38]	Spectral residual filter + Maximally Stable Extremal Regions	80.12
Garbage Detection [39]	Histogram of Oriented Gradients (HOG) + Gabor + Color	80.32
Trash detection [40]	SVM + Scale-invariant feature transform (SIFT)	63

**Table 4:** CNN Case Study.

Case Study	CNN Description	Number of Classes	Detection Accuracy	Average Detection Time
Garbage detection on grass [8]	SegNet + ResNet	5	96	8.1
Marine debris detection [41]	Faster RCNN Inception v2	3	81.0	NA
	SSD MobileNet v2	3	69.8	
	Tiny-YOLO	3	31.6	
Floor debris detection [13]	Faster RCNN ResNet	2	97.8	184.1
	Mobilenet V2 SSD		95.5	71
Trash classification [27]	11 layer CNN	6	22	NA
Proposed system	Customized 16 layer CNN	2	96	NA

**Table 5:** Performance of different models for litter detection.

CNN Network	Prec	Rec	$F_{1}$
Faster RCNN Resnet	96.9	99.4	98.1
SSD MobileNet V2	94.6	99.3	97.2
Proposed scheme	96.3	97.7	95.8

**Table 6:** Execution time.

Function	Computational Cost (Seconds) *(avg.)*
Path planning	0.0145
Motion planning	210.5

## Supplementary Materials

The following are available online at https://www.mdpi.com/1424-8220/20/6/1698/s1, Video S1:Demos of solid, liquid litters detection and the table cleaning actions by HSR.

## References

[1] The 5 Most Unwanted Jobs in Singapore. https://sg.finance.yahoo.com/news/5-most-unwanted-jobs-singapore-160000715.html.

[2] Le A., Prabakaran V., Sivanantham V., Mohan R. (2018). Modified a-star algorithm for efficient coverage path planning in tetris inspired self-reconfigurable robot with integrated laser sensor. Sensors.

[3] Le A.V., Ku P.C., Than Tun T., Huu Khanh Nhan N., Shi Y., Mohan R.E. (2018). Realization energy optimization of complete path planning in differential drive based self-reconfigurable floor cleaning robot. Energies.

[4] Kouzehgar M., Tamilselvam Y.K., Heredia M.V., Elara M.R. (2019). Self-reconfigurable façade-cleaning robot equipped with deep-learning-based crack detection based on convolutional neural networks. Autom. Constr..

[5] Ilyas M., Shi Y., Mohan R.E., Devarassu M., Kalimuthu M. (2018). Design of sTetro: A Modular, Reconfigurable, and Autonomous Staircase Cleaning Robot. J. Sens..

[6] Le A.V., Hayat A.A., Elara M.R., Nhan N.H.K., Prathap K. (2019). Reconfigurable Pavement Sweeping Robot and Pedestrian Cohabitant Framework by Vision Techniques. IEEE Access.

[7] Hayat A.A., Parween R., Elara M.R., Parsuraman K., Kandasamy P.S. Panthera: Design of a reconfigurable pavement sweeping robot. Proceedings of the 2019 International Conference on Robotics and Automation (ICRA).

[8] Bai J., Lian S., Liu Z., Wang K., Liu D. (2018). Deep Learning Based Robot for Automatically Picking Up Garbage on the Grass. IEEE Trans. Consum. Electron..

[9] The Partnership for Robotics in Europe, Robotics 2020 Multi-Annual Roadmap For Robotics in Europe Horizon 2020 Call ICT-2016 (ICT-25 & ICT-26)l. https://www.eu-robotics.net/sparc/upload/about/files/H2020-Robotics-Multi-Annual-Roadmap-ICT-2016.pdf.

[10] Yi J., Yi S. Mobile Manipulation for the HSR Intelligent Home Service Robot. Proceedings of the 2019 16th International Conference on Ubiquitous Robots (UR).

[11] Mitrevski A., Padalkar A., Nguyen M., Plöger P.G., Chalup S., Niemueller T., Suthakorn J., Williams M.A. (2019). “Lucy, Take the Noodle Box!”: Domestic Object Manipulation Using Movement Primitives and Whole Body Motion. RoboCup 2019: Robot World Cup XXIII.

[12] Park J.H., Park D.R. (2011). Dust Detection Method and Apparatus for Cleaning Robot. U.S. Patent.

[13] Ramalingam B., Lakshmanan A.K., Ilyas M., Le A.V., Elara M.R. (2018). Cascaded Machine-Learning Technique for Debris Classification in Floor-Cleaning Robot Application. Appl. Sci..

[14] Bormann R., Fischer J., Arbeiter G., Weisshardt F., Verl A. A visual dirt detection system for mobile service robots. Proceedings of the ROBOTIK 2012.

[15] Andersen N.A., Braithwaite I.D., Blanke M., Sorensen T. Combining a novel computer vision sensor with a cleaning robot to achieve autonomous pig house cleaning. Proceedings of the Decision and Control, 2005 and 2005 European Control Conference.

[16] Martínez D., Alenya G., Torras C. (2015). Planning robot manipulation to clean planar surfaces. Eng. Appl. Artif. Intell..

[17] Kabir A.M., Kaipa K.N., Marvel J.A., Gupta S.K. (2017). Automated Planning for Robotic Cleaning Using Multiple Setups and Oscillatory Tool Motions. IEEE Trans. Autom. Sci. Eng..

[18] Hess J., Sturm J., Burgard W. Learning the State Transition Model to Efficiently Clean Surfaces with Mobile Manipulation Robots. Proceedings of the Workshop on Manipulation under Uncertainty at the IEEE International Conference on Robotics and Automation (ICRA).

[19] Ramalingam B., Prabakaran V., Ilyas M., Mohan R.E., Arunmozhi M. (2018). Vision-Based Dirt Detection and Adaptive Tiling Scheme for Selective Area Coverage. J. Sens..

[20] Hess J., Beinhofer M., Kuhner D., Ruchti P., Burgard W. Poisson-driven dirt maps for efficient robot cleaning. Proceedings of the Robotics and Automation (ICRA).

[21] Lee H., Banerjee A. Intelligent scheduling and motion control for household vacuum cleaning robot system using simulation based optimization. Proceedings of the Winter Simulation Conference (WSC).

[22] Huang L., Qu H., Fu M., Deng W., Geng X., Kang B.H. (2018). Reinforcement Learning for Mobile Robot Obstacle Avoidance Under Dynamic Environments. PRICAI 2018: Trends in Artificial Intelligence.

[23] Kumar R., Kumar S., Lal S., Chand P. Object Detection and Recognition for a Pick and Place Robot. Proceedings of the Asia-Pacific World Congress on Computer Science and Engineering.

[24] Asadi K., Ramshankar H., Pullagurla H., Bhandare A., Shanbhag S., Mehta P., Kundu S., Han K., Lobaton E., Wu T. (2018). Vision-based integrated mobile robotic system for real-time applications in construction. Autom. Constr..

[25] Cheng K.P., Mohan R.E., Nhan N.H.K., Le A.V. (2019). Graph theory-based approach to accomplish complete coverage path planning tasks for reconfigurable robots. IEEE Access.

[26] Fulton M., Hong J., Islam M.J., Sattar J. (2018). Robotic Detection of Marine Litter Using Deep Visual Detection Models. arXiv.

[27] Rad M.S., von Kaenel A., Droux A., Tièche F., Ouerhani N., Ekenel H.K., Thiran J.P. (2017). A Computer Vision System to Localize and Classify Wastes on the Streets. International Conference on Computer Vision Systems.

[28] Lee J., Park G., Moon Y., Lee S., Seo T. (2019). Robust design of detecting contaminants in façade cleaning applications. IEEE Access.

[29] Zhihong C., Hebin Z., Yanbo W., Binyan L., Yu L. A vision-based robotic grasping system using deep learning for garbage sorting. Proceedings of the 36th Chinese Control Conference (CCC).

[30] Ravankar A., Ravankar A.A., Kobayashi Y., Hoshino Y., Peng C.C. (2018). Path smoothing techniques in robot navigation: State-of-the-art, current and future challenges. Sensors.

[31] Wang N., Chen C., Di Nuovo A. (2020). A Framework of Hybrid Force/Motion Skills Learning for Robots. IEEE Trans. Cogn. Dev. Syst..

[32] Yamamoto T., Terada K., Ochiai A., Saito F., Asahara Y., Murase K. (2019). Development of Human Support Robot as the research platform of a domestic mobile manipulator. ROBOMECH J..

[33] Hidayatullah A.S., Jati A.N., Setianingsih C. Realization of depth first search algorithm on line maze solver robot. Proceedings of the 2017 International Conference on Control, Electronics, Renewable Energy and Communications (ICCREC).

[34] Raheem F., Abdulkareem M. (2019). Development of Path Planning Algorithm Using Probabilistic Roadmap Based on Modified Ant Colony Optimization. World J. Eng. Technol..

[35] YOLO Machine Learning. https://scholar.google.ca/scholar?hl=en&as_sdt=0,5&q=YOLO+machine+learning#d=gs_qabs&u=%23p%3DWYCdg0zDi5EJ.html.

[36] Quigley M., Conley K., Gerkey B., Faust J., Foote T., Leibs J.R., Wheeler R., Ng A.Y. ROS: An open-source Robot Operating System. Proceedings of the ICRA Workshop on Open Source Software.

[37] Chitta S., Sucan I., Cousins S. (2012). Moveit![ros topics]. IEEE Rob. Autom Mag..

[38] Milinda H., Madhusanka B. Mud and dirt separation method for floor cleaning robot. Proceedings of the Engineering Research Conference (MERCon).

[39] Mittal G., Yagnik K.B., Garg M., Krishnan N.C. Spotgarbage: smartphone app to detect garbage using deep learning. Proceedings of the 2016 ACM International Joint Conference on Pervasive and Ubiquitous Computing.

[40] Yang G.T.M., Thung G. (2016). Classification of Trash for Recyclability Status. CS229 Project Report. https://pdfs.semanticscholar.org/c908/11082924011c73fea6252f42b01af9076f28.pdf.

[41] Valdenegro-Toro M. Submerged marine debris detection with autonomous underwater vehicles. Proceedings of the Robotics and Automation for Humanitarian Applications (RAHA).

[42] Sandler M., Howard A., Zhu M., Zhmoginov A., Chen L.C. (2018). MobileNetV2: Inverted Residuals and Linear Bottlenecks. arXiv.

